# Post-acute COVID-19 symptom risk in hospitalized and non-hospitalized COVID-19 survivors: A systematic review and meta-analysis

**DOI:** 10.3389/fpubh.2023.1112383

**Published:** 2023-02-16

**Authors:** Niu Yuan, Zhang-Hong Lv, Chun-Rong Sun, Yuan-Yuan Wen, Ting-Yu Tao, Dan Qian, Fang-Ping Tao, Jia-Hui Yu

**Affiliations:** ^1^Department of Nursing, The First Affiliated Hospital of Zhejiang University School of Medicine, Hangzhou, China; ^2^Department of Respiratory Medicine, The First Affiliated Hospital of Zhejiang University School of Medicine, Hangzhou, China; ^3^Ear, Nose and Throat Department, The First Affiliated Hospital of Zhejiang University School of Medicine, Hangzhou, China; ^4^Department of Surgical Oncology, The First Affiliated Hospital of Zhejiang University School of Medicine, Hangzhou, China

**Keywords:** post-acute COVID-19 symptom, long-COVID, hospitalized, non-hospitalized, COVID-19 survivor, meta-analysis

## Abstract

**Background:**

Post-acute coronavirus disease 2019 (COVID-19) symptoms occurred in most of the COVID-19 survivors. However, few studies have examined the issue of whether hospitalization results in different post-acute COVID-19 symptom risks. This study aimed to compare potential COVID-19 long-term effects in hospitalized and non-hospitalized COVID-19 survivors.

**Methods:**

This study is designed as a systematic review and meta-analysis of observational studies. A systematic search of six databases was performed for identifying articles published from inception until April 20th, 2022, which compared post-acute COVID-19 symptom risk in hospitalized and non-hospitalized COVID-19 survivors using a predesigned search strategy included terms for SARS-CoV-2 (eg, *COVID, coronavirus*, and *2019-nCoV*), post-acute COVID-19 Syndrome (eg, *post-COVID, post COVID conditions, chronic COVID symptom, long COVID, long COVID symptom, long-haul COVID, COVID sequelae, convalescence*, and *persistent COVID symptom*), and hospitalization (*hospitalized, in hospital*, and *home-isolated*). The present meta-analysis was conducted according to The Preferred Reporting Items for Systematic Reviews and Meta-Analyses (PRISMA) 2020 statement using R software 4.1.3 to create forest plots. Q statistics and the *I*^2^ index were used to evaluate heterogeneity in this meta-analysis.

**Results:**

Six observational studies conducted in Spain, Austria, Switzerland, Canada, and the USA involving 419 hospitalized and 742 non-hospitalized COVID-19 survivors were included. The number of COVID-19 survivors in included studies ranged from 63 to 431, and follow-up data were collected through visits in four studies and another two used an electronic questionnaire, visit and telephone, respectively. Significant increase in the risks of long dyspnea (OR = 3.18, 95% CI = 1.90–5.32), anxiety (OR = 3.09, 95% CI = 1.47–6.47), myalgia (OR = 2.33, 95% CI = 1.02–5.33), and hair loss (OR = 2.76, 95% CI = 1.07–7.12) risk were found in hospitalized COVID-19 survivors compared with outpatients. Conversely, persisting ageusia risk was significantly reduced in hospitalized COVID-19 survivors than in non-hospitalized patients.

**Conclusion:**

The findings suggested that special attention and patient-centered rehabilitation service based on a needs survey should be provided for hospitalized COVID-19 survivors who experienced high post-acute COVID-19 symptoms risk.

## 1. Introduction

COVID-19 is an infectious disease caused by severe acute respiratory syndrome coronavirus 2 (SARS-CoV-2), which has the characteristics of strong contagion and high mortality (1, 2). As of May 16th, 2022, there have been 519 million cumulative confirmed cases resulting from the COVID-19 pandemic according to the live world statistics released by the World Health Organization. With the continuing growth of infected cases across the world, the complication and sequelae of COVID-19 have gradually attracted the attention of health professionals (3). The sequelae of COVID-19 can last several weeks or months, which is also called the post-acute COVID-19 symptom (PACS). Evidence shows that SARS-CoV-2 infection causes direct damage to multi-organs and one or more organ impairments presented in almost 70% of 201 patients (4). Alkodaymi et al. (5) have conducted a systematic review and meta-analysis of 63 studies and 257, 348 COVID-19 survivors and stated most of the patients experienced PACS for at least 3 months after recovery from COVID-19 infection. A cross-sectional observational study by Tabacof et al. (6) investigated the influences of PACS on physical and cognitive function, quality of life, and usual activity, which found that PACS had a multifaceted impact on the lives of 156 COVID-19 survivors almost one year after infection.

SARS-CoV-2 can cause various degrees of damage to the respiratory system and extrapulmonary organs such as the immune system, digestive system, cardiovascular system, and nervous system (7, 8). Even if the clinical symptoms of patients disappear, most of the patients still have sequelae (9). The number of estimated PACSs after initial recovery can be reached 55 in 80% of 47, 910, and the five frequently reported PACSs were dyspnea, headache, fatigue, attention disorder, and hair loss according to the systematic review and meta-analysis by Lopez-Leon et al. (10). Presently, the prevalence of PACSs has been widely investigated, while the risk factors provoking the development of PACSs have been little studied. A systematic review and meta-analysis by Maglietta et al. (11) stated that female sex and acute disease severity were two risk factors for the development of one or more PACSs. However, the development of PACSs is still not yet comprehensively studied as it affects COVID-19 survivors who are not hospitalized. A cohort study by Petersen et al. (12) assessed the multi-organ functions between non-hospitalized populations after SARS-CoV-2 infection and healthy individuals and stated that the subclinical multi-organ affection signs related to respiratory, cardiovascular, vascular, and renal system organs were identified. In another cohort study comprising 176 hospitalized and 72 non-hospitalized individuals after SARS-CoV-2 infection, PACSs were more frequently presented in hospitalized COVID-19 survivors (13). But the loss of taste was more commonly presented in non-hospitalized COVID-19 survivors (14).

The data of these published studies just focused on some PACSs risk analysis, which did not provide a comprehensive analysis between hospitalized and non-hospitalized non-hospitalized.

Therefore, we conducted the present systematic review and meta-analysis to compare PACSs risk between hospitalized and non-hospitalized patients for providing the theoretical basis to develop PACS rehabilitation services.

## 2. Materials and methods

The present study was designed to compare post-acute COVID-19 symptom risk in hospitalized and non-hospitalized COVID-19 survivors. We conducted the present meta-analysis following The Preferred Reporting Items for Systematic Reviews and Meta-Analyses (PRISMA) 2020 statement, checked and confirmed that the recommended items of the PRISMA 2020 statement were reported in the present meta-analysis (15).

### 2.1. Literature search

A comprehensive search of six databases containing PubMed, Web of Science, The Cochrane Library, EMBASE, Google Scholar, and Scopus was independently performed by two co-authors from inception until April 20th, 2022. Reference lists of included studies were also manually checked to find the eligible studies as a supplement (16). The search strategy for the present meta-analysis was developed using the MeSH terms containing “SARS-CoV-2”, “post-acute COVID-19 Syndrome”, and “hospitalization” combined with the free-text terms. The terms in the process of literature search were “COVID”, “SARS-CoV-2”, “coronavirus” or “2019-nCoV” in combination with “post-acute COVID-19 symptom”, “post-acute COVID-19 symptoms”, “post-COVID”, “post COVID conditions” “chronic COVID symptom”, “chronic COVID symptoms”, “long COVID”, “long COVID symptom”, “long COVID symptoms”, “long-haul COVID”, “COVID sequalae”, “convalescence”, or “persistent COVID symptoms” and “hospitalized”, “Hospitalization”, “in hospital” and “home-isolated”. Studies published were searched with no language restriction.

### 2.2. Inclusion and exclusion criteria

To be included in the present meta-analysis there were a set of inclusion criteria that studies would be required to fulfill. The inclusion criteria were specified as follows: (1) population: included subjects who suffered SARS-CoV-2 that diagnosed by polymerase chain reaction (PCR) testing and followed up for at least 12 weeks; (2) Intervention: hospitalization as the baseline exposure; (3) Comparison: non-hospitalized COVID-19 survivors as a control; (4) Outcomes: reported PACS prevalence and the corresponding 95% confidence intervals of hospitalized COVID-19 survivors relative to those non-hospitalized; (5) Study design: prospective or retrospective observational studies which have no restriction of sample size. For multiple results obtained by observing the same participants over different lengths of time, only the latest published results with the longest observation period were included. The exclusion criteria were: (1) studies with a follow-up period of fewer than 12 weeks; (2) reviews, meta-analyses, case reports, conference abstracts, or animal trials; (3) studies without complete data for data synthesis; (4) duplicated articles or data. For multiple results obtained by observing the same participants over different lengths of time, only the latest published results with the longest observation period were included.

### 2.3. Literature screening

The retrieved records of four databases that fulfilled the eligibility criteria were downloaded and independently screened by two co-authors who contributed to the literature screening. In case of disagreement, a consensus would be reached through discussion. If no consensus could be reached, the decision would be made by re-review of another two co-authors. During literature screening, the titles and abstracts were first to be read to exclude irrelevant studies. The full texts were further read to determine whether to include the study.

### 2.4. Data extraction

The data were systematically abstracted from included study containing the first author's family name, year of publication, study design, the country where the study was carried out, number and age of included COVID-19 survivors, follow-up mode and length, symptoms of PACS, and effect estimates of prevalence. Data extraction in the present meta-analysis was conducted by two co-authors. In case of discrepancies, a discussion is needed, and when necessary, the decision is reached by another two co-authors.

### 2.5. Quality assessment

The quality assessment of the included studies was carried out by two co-authors using the Newcastle-Ottawa Scale (17), which was widely used in the previous systematic review for prospective and retrospective observational studies on the topic of COVID-19 (18, 19). This nine-point scale consists of 8 items in three dimensions: selection, comparability, and outcome. Selection, comparability, and outcome of cohorts containing 4, 1, and 3 items with 4, 2, and 3 scores respectively. In case of discrepancies, a discussion is needed, and when necessary, the decision is reached by another two co-authors.

### 2.6. Statistical analysis

To compare PACS risk between hospitalized and non-hospitalized COVID-19 survivors, the odds ratios (ORs) and 95% confidence intervals (CIs) were calculated with raw data using *R* software 4.1.3. Heterogeneity among studies was assessed using Cochrane's *Q*-test (with *P* > 0.1 indicating heterogeneity) and the *I*^2^ statistic (where *I*^2^ ≥ 50% indicated significant heterogeneity). The random or fixed effects model was chosen and applied for data synthesis according to the results of heterogeneity. Sensitivity analyses by removing studies one at a time were conducted for all indicators to evaluate the stability of pooled results. Publication bias was assessed using funnel plots (asymmetrical funnel plot indicated a significant publication bias) and Egger's test (with *P* < 0.05 indicated a significant publication bias).

## 3. Results

### 3.1. Study selection

**Figure 1:**
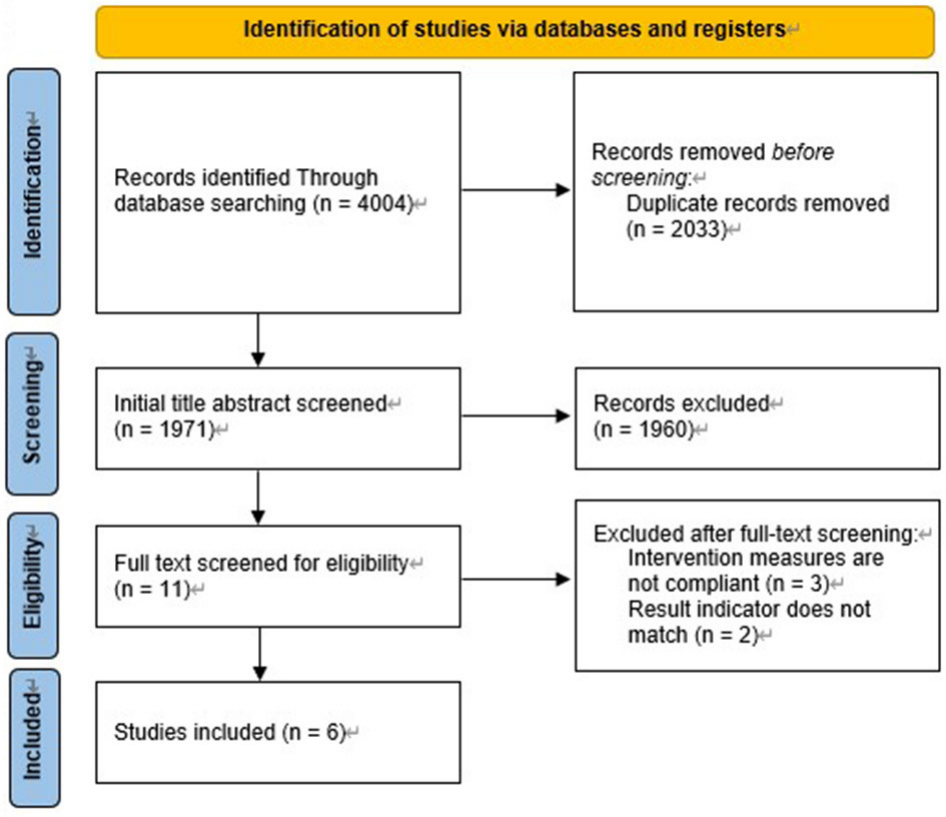
Flowchart of the database search and study selection.

A total of 4004 records were yielded from searching six databases, by which 2,033 duplicated studies were excluded. After screening the title and abstract of the remaining 1,971 records, 11 potentially included studies identified for full-text review. Three studies were excluded because intervention measures are not compliant (20–23); another two studies were excluded because the result indicator does not match (24, 25). In the last, a total of 6 studies were included in the present meta-analysis (Figure 1).

### 3.2. Study characteristics

The characteristics of all six included observational studies conducted between 2021 and 2022 were shown in Table 1. The included studies were set in Spain (13), Austria (14), Switzerland (26), Canada (27), and the USA (28, 29). The number of COVID-19 survivors in included studies ranged from 63 to 431, and the prevalence of PACS was prospectively recorded. Follow-up was carried out through visits in four studies (14, 27–29), and another two used an electronic questionnaire (26), visit and telephone (13) respectively. The results of the quality assessment showed that there were three (13, 27, 29), two (14, 28), and one (26) studies scored 6, 7, and 8 points respectively, which indicated that the quality of included studies is good with low risk.

**Table 1:** The characteristics of included studies.

**First Author**	**Study Design**	**Country**	**Age (Years)**	**Sample size (M/F)**	**Hospitalized(M/F)**	**Non-Hospitalized(M/F)**	**Follow-Up Length (Months)**	**Follow-Up Mode**
Pérez-González et al. (13)	Prospective cohort study,	Spain	Median (IQR) of Hospitalized: 62 (51–71) Median (IQR) of Non-Hospitalized: 47 (34-54)	248 (148/100)	172 (103/69)	76 (45/31)	6	Visit and Telephone
	single center							
Rass et al. (14)	Prospective cohort study, multicenter	Austria	Median (IQR) of Hospitalized: 56 (48–68)	135 (82/53)	103 (72/31)	32 (10/22)	3	Visit
Menges et al. (26)	Prospective cohort study, single center	Switzerland	Median (IQR): 47 (33-58)	431 (217/214)	81	350	8	Electric questionnaire
Abdallah et al. (27)	Prospective cohort study, single center	Canada	Mean (SD) of Hospitalized: 59.1 ± 13.5 Mean (SD) of Non-hospitalized: 42.4 ± 12.9	63 (36/27)	25 (16/9)	38 (20/18)	4	Visit
Jacobson et al. (28)	Prospective cohort study, single center	USA	Mean (SD) of Hospitalized: 50.6 ± 15.1 Mean (SD) of Non-hospitalized: 41.6 ± 12.5	118 (49/47)	22 (14/8)	96 (49/47)	3-4	Visit
Logue et al. (29)	Prospective cohort study, single center	USA	Mean (SD) of Hospitalized: 54 ± 15.1 Mean (SD) of Non-hospitalized: 46.3 ± 14.3	166 (76 /101)	16 (8/8)	150 (63/87)	3-9	Visit

### 3.3. PACS risk among Hospitalized vs. non-hospitalized COVID-19 survivors

#### 3.3.1. Any persistent symptom risk

**Figure 2:**
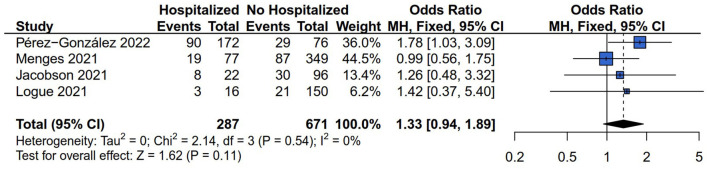
Forest plot for meta-analysis of any persistent symptom risk among Hospitalized *vs*. non-hospitalized COVID-19 survivors.

The data of any persistent symptom with 963/1,161 (82.95%) patients were extracted from four studies (13, 26, 28, 29) for data synthesis. In these included studies, a total of 287 and 671 subjects were grouped into hospitalized and non-hospitalized groups. The fixed model was applied because there was no heterogeneity difference according to the results of *Q* statistics and the *I*^2^ index (*I*^2^ = 0%*, P* = 0.54). The results of data synthesis showed that there was no difference in the outcome of any persistent symptom risk between hospitalized and non-hospitalized COVID-19 survivors (OR = 1.33, 95% CI: 0.94 to 1.89, *P* = 0.11) (Figure 2).

#### 3.3.2. General symptom risk (fatigue)

**Figure 3:**
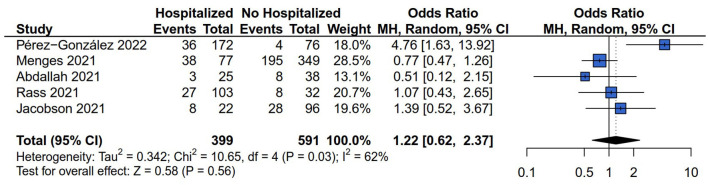
Forest plot for meta-analysis of general symptom risk among Hospitalized *vs*. non-hospitalized COVID-19 survivors.

Five (13, 14, 26–28) of the six studies reported the general symptom risk of fatigue involving 995/1,161 (85.70%) cases, and 40.50% hospitalized COVID-19 survivors (403/995). The prevalence of fatigue was 28.07% in hospitalized COVID-19 survivors (112/399) and 41.12% in non-hospitalized COVID-19 survivors (243/591). The random model was applied because the result of the *Q* statistics and *I*^2^ index indicated that the heterogeneity was significant (*I*^2^ = 62%*, P* = 0.03). The results showed no difference in the outcome of fatigue risk between hospitalized and non-hospitalized COVID-19 survivors (OR = 1.22, 95% CI: 0.62–2.37, *P* = 0.56) (Figure 3).

#### 3.3.3. Respiratory symptoms risk (dyspnea, cough, and chest pain)

**Figure 4:**
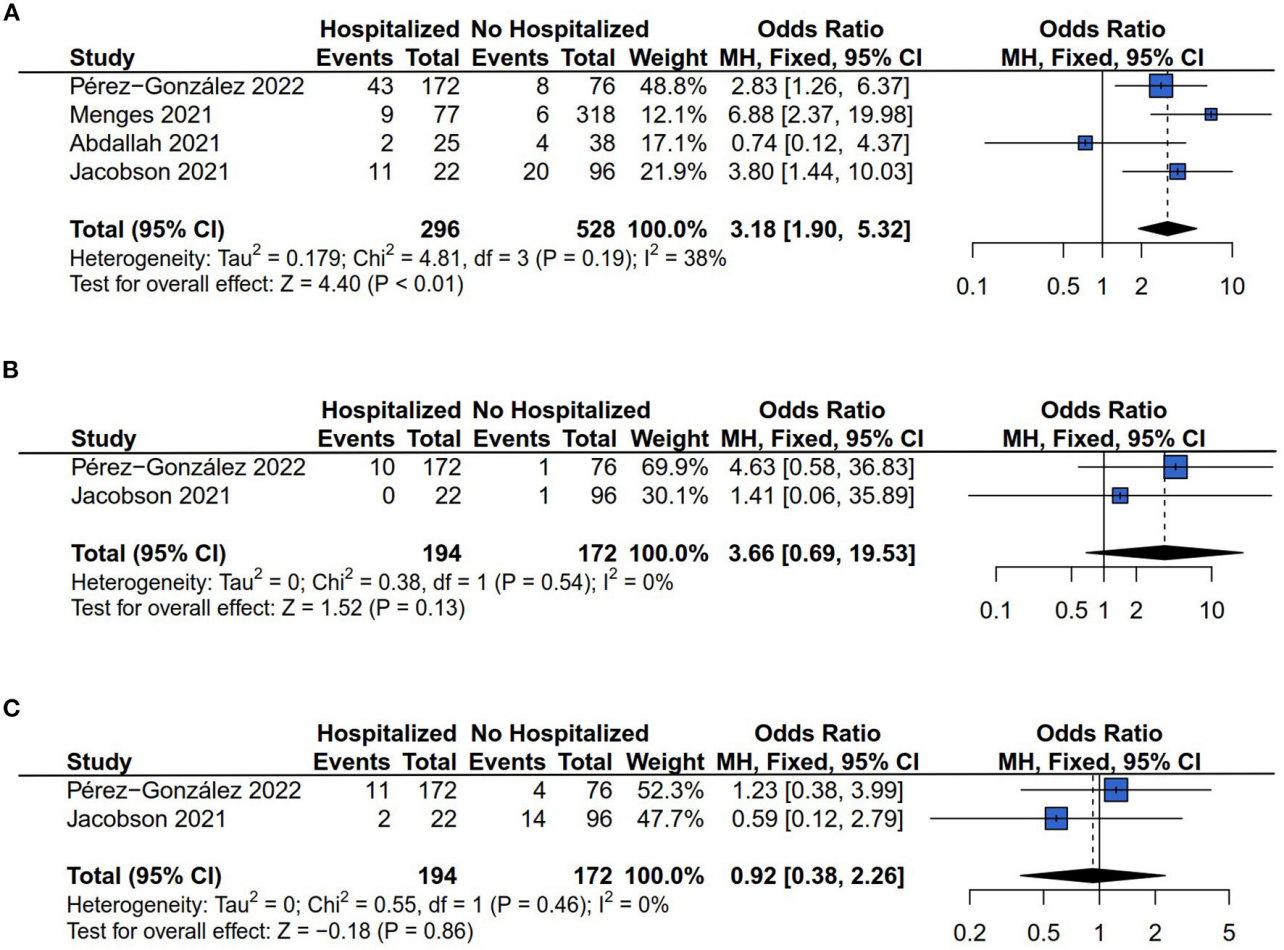
Forest plot for meta-analysis of respiratory symptoms risk among Hospitalized *vs*. non-hospitalized COVID-19 survivors. **(A)** Dyspnea risk; **(B)** Cough risk; **(C)** Chest pain risk.

There were four (13, 26–28), two (13, 28), and two (13, 28) of the six studies that reported the dyspnea, cough, and chest pain risk respectively. The results of *Q* statistics and *I*^2^ index showed no heterogeneity of effect size in the comparison of dyspnea (*I*^2^ = 38%*, P* = 0.19), cough (*I*^2^ = 0%*, P* = 0.54), and chest pain (*I*^2^ = 0%*, P* = 0.46) risk between hospitalized and non-hospitalized COVID-19 survivors. Figure 4A showed that hospitalization increased the patients' persistent dyspnea risk compared with non-hospitalized COVID-19 survivors (OR = 3.18, 95% CI: 1.90 to 5.32, *P* < 0.01). However, the comparison results between hospitalized and non-hospitalized COVID-19 survivors indicated no difference in the outcome of cough (OR = 3.66, 95% CI: 0.69–19.53, *P* = 0.13) (Figure 4B) and chest pain risk (OR = 0.92, 95% CI: 0.38–2.26, *P* = 0.86) (Figure 4C).

#### 3.3.4. Neurological symptoms risk (headache, sleep disorder, ageusia, anosmia, anxiety, and depression)

**Figure 5:**
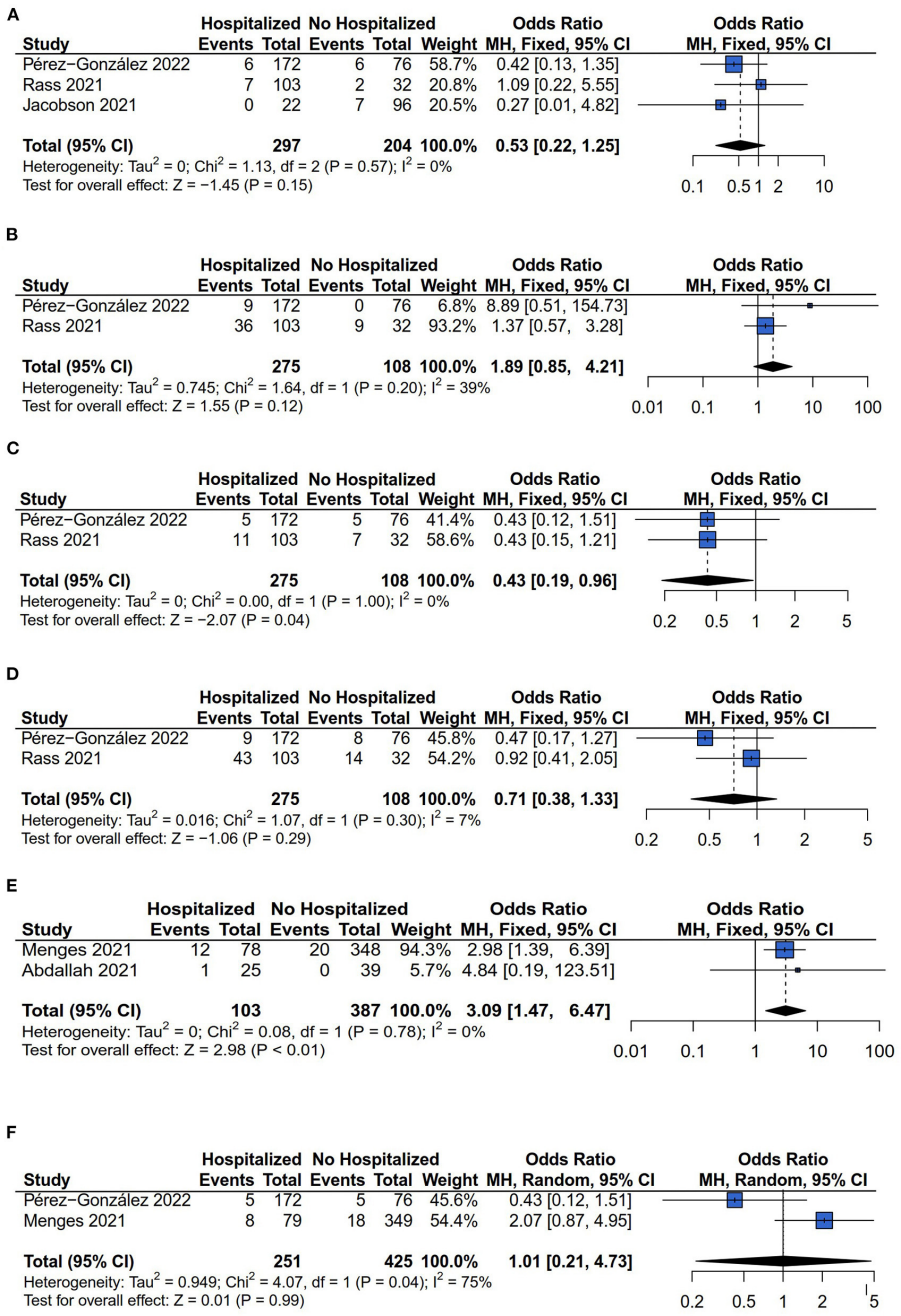
Forest plot for meta-analysis of neurological symptoms risk among Hospitalized *vs*. non-hospitalized COVID-19 survivors. **(A)** Headache risk; **(B)** Sleep disorder risk; **(C)** Ageusia risk; **(D)** Anosmia risk; **(E)** Anxiety risk; **(F)** Depression risk.

Six types of neurological symptoms were investigated between hospitalized and non-hospitalized COVID-19 survivors: Headache, sleep disorder, ageusia, anosmia, anxiety, and depression. For the outcome of headache, sleep disorder, ageusia, anosmia, anxiety, and depression, the comparisons between hospitalized and non-hospitalized COVID-19 survivors were investigated by three (13, 14, 28), two (13, 14), two (13, 14), two (13, 14), two (26, 27), and two (13, 26) studies respectively. The fixed models have been applied for analyzing the comparison of headache, sleep disorder, ageusia, anosmia, and anxiety risk because there was no heterogeneity difference (headache: *I*^2^ = 0%, *P* = 0.57; sleep disorder: *I*^2^ = 39%, *P* = 0.20; ageusia: *I*^2^ = 0%, *P* = 1.00; anosmia: *I*^2^ = 7%, *P* = 0.30; anxiety: *I*^2^ = 0%, *P* = 0.78). While the random model was applied for comparing the depression risk because of heterogeneity difference (*I*^2^ = 75%, *P* = 0.04). The results showed that the hospitalization decreased the patients' persistent ageusia risk compared with non-hospitalized COVID-19 survivors (OR = 0.43, 95% CI: 0.19–0.96, *P* = 0.04) (Figure 5C). Conversely, the anxiety risk was increased in hospitalized COVID-19 survivors compared with non-hospitalized COVID-19 survivors (OR = 3.09, 95% CI: 1.47– 6.47, *P* < 0.01) (Figure 5E). However, the significant differences in headache (OR = 0.53, 95% CI: 0.22–0.25, *P* = 0.15) (Figure 5A), sleep disorder (OR = 1.89, 95% CI: 0.85–4.21, *P* = 0.12) (Figure 5B), anosmia (OR = 0.71, 95% CI: 0.38–1.33, *P* = 0.29) (Figure 5D), and depression (OR = 1.01, 95% CI: 0.21–4.73, *P* = 0.99) (Figure 5F) risk has not been found between hospitalized and non-hospitalized COVID-19 survivors.

#### 3.3.5. Other symptoms risk (myalgia and hair loss)

**Figure 6:**
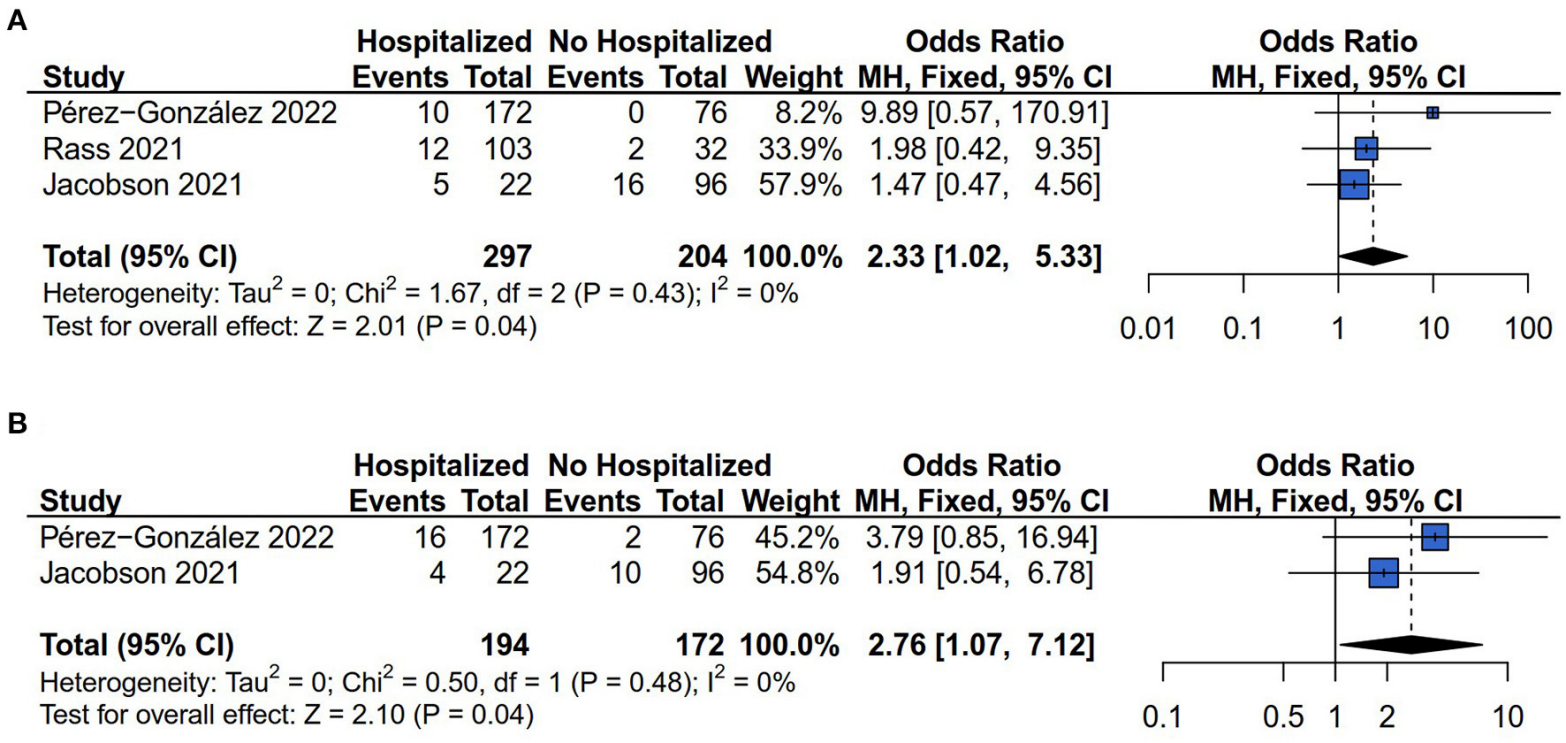
Forest plot for meta-analysis of other symptoms risk among Hospitalized *vs*. non-hospitalized COVID-19 survivors. **(A)** Myalgia risk; **(B)** Hair loss risk.

There were three (13, 14, 28) and two (13, 28) of the six studies that examined the myalgia and hair loss risk respectively. The results of Cochrane's *Q*-test and the *I*^2^ statistic showed no heterogeneity of effect size in the comparison of myalgia (*I*^2^ = 0%*, P* = 0.43) and hair loss (*I*^2^ = 0%*, P* = 0.48) risk between hospitalized and non-hospitalized COVID-19 survivors. The results showed that the association between hospitalization and myalgia (OR = 2.33, 95% CI: 1.02–5.33, *P* = 0.04) (Figure 6A), and hair loss (OR = 2.76, 95% CI: 1.07 to 7.12, *P* = 0.04) (Figure 6B) risk among COVID-19 survivors.

### 3.4. Sensitivity analysis and publication bias

The stability of the pooled result was assessed by sensitivity analysis. The results of sensitivity analysis of OR for PACSs risk comparing hospitalized and non-hospitalized COVID-19 survivors showed no significant heterogeneity (Supplementary Figures 1A–L). The publication bias of the pooled result was evaluated by constructing funnel plots and performing Egger's test when the number of included studies was equal to or more than 3. The results of funnel plots and Egger's test suggested little evidence of publication bias for any persistent symptom, fatigue, dyspnea, headache, and myalgia risk comparing hospitalized and non-hospitalized COVID-19 survivors (Supplementary Figures 2A–E).

## 4. Discussion

The present systematic review and meta-analysis summarized the association of hospitalization and PACSs among COVID-19 survivors from six studies. These six studies were of moderate to high quality. Because the pooled risk generated was based on crude estimates, these findings need to be interpreted with caution. Significant differences in some PACS risks were observed from hospitalized compared with non-hospitalized COVID-19 survivors, but not all. The results of this systematic review and meta-analysis showed that significant increase in the risk of long dyspnea (OR = 3.18, 95% CI = 1.90–5.32), anxiety (OR = 3.09, 95% CI = 1.47–6.47), myalgia (OR = 2.33, 95% CI = 1.02–5.33), and hair loss (OR = 2.76, 95% CI = 1.07–7.12) risk was found in hospitalized COVID-19 survivors compared with outpatients.

Evidence on why PACSs occur is still largely unknown, however, numerous studies have confirmed that PACSs developed regardless of the initial disease severity (30). Respiratory symptoms lasting over 28 days were commonly reported among 4,182 COVID-19 survivors in a prospective cohort study (31). A multicenter cross-sectional study by Mandal et al. (32) stated that CT lung abnormalities were found among 38% of COVID-19 survivors after discharge from the hospital at a median follow-up time of 54 days. Another prospective study carried out by Vijayakumar et al. (33) also investigated the imaging abnormalities in COVID survivors after hospital discharge at a longer median follow-up time of 105 days and found that 56% of patients presented with persistent CT lung abnormalities mainly characterized as ground-glass opacification and irregular bands. Whist lung impairment was another concern among COVID-19 survivors. A systematic review and meta-analysis conducted by Torres-Castro et al. (34) summarized the prevalence of lung impairment in COVID-19 survivors and found that diffusion capacity was predominating. The influencing factors for diffusion impairment at 180 days after hospital discharge were female sex, age, and peak RALE score (35). Dyspnea, cough, and chest pain have been proven to be the predominant persistent respiratory symptoms (36). Our findings indicated a significant increase in dyspnea risk among hospitalized COVID-19 survivors compared with non-hospitalized. A multicenter prospective study by Bretas et al. (37) demonstrated that the prevalence of dyspnea was as high as 64.7% in ward admission. The potential reason why high dyspnea risk presented in hospitalized COVID-19 survivors compared with non-hospitalized is COVID-induced persistent abnormality within the microstructure of the lungs or in the pulmonary vasculature (38).

Significant differences in neurological symptoms (ageusia and anxiety) risk between hospitalized and non-hospitalized were found in this meta-analysis. According to a cross-sectional study by Sampaio Rocha-Filho et al. (39), ageusia was a dominant persistent neurological symptom among 288 COVID-19 survivors with a high occurrence rate of 69.8%. However, another prospective follow-up study by Nielsen et al. (40) stated that the prevalence of long-lasting ageusia was highly increased among mild COVID-19 survivors, which is consistent with our findings that persisting ageusia risk is significantly decreased in hospitalized individuals compared with non-hospitalized populations after SARS-CoV-2 infection. This above result might be associated with the pathological basis of neurotropic infection in the gustatory system (41). A meta-analysis of 19 studies has demonstrated that anxiety was a frequent neuropsychiatric manifestation among COVID-19 survivors (42). Whist, anxiety was a common persistent neurological symptom among most of the non-hospitalized COVID-19 survivors (43). Our meta-analysis revealed a high prevalence of anxiety in hospitalized compared with non-hospitalized COVID-19 survivors. The mechanism related to anxiety risk include systemic inflammation response to SARS-CoV-2 infection and perceived stress before and during COVID-19 infection (44).

High risks of other symptoms containing myalgia and hair loss have been identified among hospitalized compared with non-hospitalized COVID-19 survivors in the present meta-analysis. A previous scoping review by Cha et al. (45) stated that myalgia was a commonly reported PACS in non-hospitalized COVID-19 survivors. But, the previous studies did not compare the prevalence of myalgia between hospitalized and non-hospitalized COVID-19 survivors (24, 46–48). Persisting myalgia risk was higher among hospitalized COVID-19 survivors than non-hospitalized ones based on the present quantitative meta-analysis. A hypothesis for the mechanism proposed by Kucuk et al. (49) said that muscle pain might be associated with increased lactate levels resulting from both elevated lactate dehydrogenase and anaerobic glycolysis. Hair loss has been reported as a frequently persistent symptom among COVID-19 survivors (50). A preliminary study by Goren et al. (51) stated that androgen expression might be related to the severity of COVID-19 infection among hospitalized COVID-19 patients with male pattern hair loss. A retrospective study by Sunada et al. (52) demonstrated the relationship between hair loss and hormone trends, which might be the reason for the significant difference in hair loss risk between hospitalized and non-hospitalized COVID-19 survivors.

The present systematic review and meta-analysis is the first time to compare PACSs risk in hospitalized and non-hospitalized COVID-19 survivors. However, there are several limitations existed in this study. Firstly, the included studies have a restriction on the published language of Chinese or English. Secondly, five of the six studies were single-center prospective studies with limited sample sizes. Thirdly, heterogeneity differences in the outcomes of fatigue and depression risks of the included studies were another crucial limitation in this meta-analysis. Finally, the PACS risks were compared between hospitalized and non-hospitalized COVID-19 survivors at different lengths of follow-up time, which may significantly influence the results.

## 5. Conclusion

In conclusion, the present meta-analysis has provided a comprehensive analysis of PACSs risk between hospitalized and non-hospitalized COVID-19 survivors. It showed that those in hospitalization experienced high post-acute COVID-19 symptoms risk such as dyspnea, anxiety, myalgia, and hair loss, and a low risk of persisting ageusia. Health professionals should pay special attention to PACSs risk for hospitalized COVID-19 survivors and provide patient-centered rehabilitation services. Moreover, Health professionals could develop assessment tools of high quality to evaluate persisting dyspnea, ageusia, anxiety, myalgia, and hair loss risk, especially for hospitalized COVID-19 survivors. Meanwhile, the need for a patient-centered strategy for long-COVID care is urgent that should be investigated.

## Data availability statement

The original contributions presented in the study are included in the article/Supplementary material, further inquiries can be directed to the corresponding author.

## Author contributions

All authors listed have made a substantial, direct, and intellectual contribution to the work and approved it for publication.
